# Prevalence of risk for pressure ulcers, malnutrition, poor oral health and falls – a register study among older persons receiving municipal health care in southern Sweden

**DOI:** 10.1186/s12877-021-02205-x

**Published:** 2021-04-21

**Authors:** Merita Neziraj, Peter Hellman, Christine Kumlien, Magdalena Andersson, Malin Axelsson

**Affiliations:** 1 Department of Care Science, Faculty of Health and Society, Malmö University, Jan Waldenströms Gata 25, SE-20506 Malmö, Sweden; 2 Department of Cardio-Thoracic and Vascular Surgery, Skåne University Hospital, Malmö, Sweden; 3 Department of Health and Social Care, Strategic Development, Unit of Research, Quality and Education, Malmö Stad, Kungsgatan 13, SE-20580 Malmö, Sweden

**Keywords:** Falls, Malnutrition, Municipal health care, Older persons, Poor oral health, Pressure ulcers, Prevention, Register study, Risk assessment, Senior alert

## Abstract

**Background:**

Although pressure ulcers, malnutrition, poor oral health and falls are common among older persons, causing deteriorated health status, they have not been studied altogether among older persons receiving different types of municipal health care. The aim of this study was to determine the prevalence of risk for pressure ulcers, malnutrition, poor oral health and falls among older persons aged ≥65 years receiving municipal health care in southern Sweden.

**Methods:**

A retrospective cross-sectional study (*n* = 12,518 persons aged ≥65 years) using data from the national quality registry Senior Alert was conducted. The prevalence of risk for pressure ulcers, malnutrition, poor oral health and falls was calculated based on categorical data from the instruments available in Senior Alert. T-tests, chi-square test, the Mantel- Haenszel test and logistic regression models were performed.

**Results:**

The prevalence of risk for pressure ulcers, malnutrition, poor oral health and falls was 27.9, 56.3, 34.2 and 74.5% respectively. Almost 90% of the older persons had at least one health risk. The prevalence of risk for pressure ulcers, poor oral health and falls was significantly higher in dementia care units compared to short term nursing care, home health care and nursing homes. The prevalence of risk for malnutrition was significantly higher among older persons staying in short term nursing care compared to other types of housing. The odds of having a risk for malnutrition were higher in short term nursing care compared to other types of housing. The oldest age group of 95–106 years had the highest odds of having a risk for falls. The presence of multiple health risks in one subject were more common in dementia homes compared to nursing homes and home health care but not compared to short term nursing care.

**Conclusion:**

The prevalence of risk for pressure ulcers, malnutrition, poor oral health and falls was high, implying that these health risks are a great concern for older persons receiving municipal health care. A comprehensive supporting preventive process to prevent all the investigated health risks among older persons receiving municipal health care is recommended.

**Supplementary Information:**

The online version contains supplementary material available at 10.1186/s12877-021-02205-x.

## Background

Pressure ulcers, malnutrition, poor oral health and falls are all related and common among frail older persons aged 65 years and older [1] and, increase the risk of disability, hospitalization, nursing home admission [2] and substantial healthcare costs [3]. Because the causes of pressure ulcers, malnutrition, poor oral health and falls are multifactorial and related to frailty [4], a prevalence study reflecting these health risks altogether will contribute to a more complete picture, which may improve knowledge in the field of preventive work with older persons.

Pressure ulcers are a worldwide problem in health care settings [5]. The prevalence of pressure ulcers varies internationally from 4.3–30.8% [6–11] and in Sweden, the prevalence varies between 11.8 and 14.5% in nursing homes [11]. Age, general health status, immobility [12], female sex [13] and malnutrition [14] are risk factors for pressure ulcers. Pressure ulcers are also associated with malnutrition, as it impairs immune and hormonal function and causes skin changes, thus increasing vulnerability to pressure ulcers [14]. Furthermore, poor oral health status [15, 16], increased risk for falls [17], increased hospitalization rates and lower survival rates [18] are severe consequences observed among malnourished older persons. Internationally, from 15 to 40% of older persons in nursing homes were at risk of malnutrition [19–21] and in Sweden, 40.3% of older persons were at risk of malnutrition, and 17.7% were assessed as malnourished [18]. The prevalence of malnutrition is increasing, emphasizing the importance of frequently assessing nutritional status to prevent malnutrition in this group of people [18].

The correlation between oral health problems and nutritional status is well underpinned, indicating the importance of evaluating oral health status in older persons with nutritional problems [22]. Aging, physical changes, along with general diseases and medications increase the risk of poor oral health [14]. Approximately 29% of older persons in a Swedish context were found to have moderate oral health problems and 12% were found to have severe problems [22]. Globally, oral health among older persons is considered a public health issue, and preventive strategies are recommended to improve the oral health of older persons [23].

Older persons in nursing homes fall more often (30–50%) than those who are living in the community [24]. Falls are the most common cause of accidents, resulting in hospitalization [25] and leading to morbidity and mortality among older persons; thus, falls are a major public health problem [26]. Falls among older persons aged 65 years and older account for 87% of all fractures in the United States [27], and in Sweden, falls among older persons cause more deaths, hospital admissions and emergency unit visits than any other type of accident [28].

The number of older persons will double from 2017 to 2050, and the largest increase is expected in the age group of individuals 80 years or older [29]. This large number of older persons will require those who work with them to have specific knowledge and competence to decrease debilitating and costly health care problems [29, 30]. In Sweden, there is a national web-based quality registry, Senior Alert [31]. The users are healthcare personnel and among the available instruments in Senior Alert, risk assessments and registrations in Senior Alert are usually done by registered nurses or nursing assistants. Senior Alert provides an individualized, standardized, structured and systematic preventive care process for persons aged 65 years or older at risk for pressure ulcers, malnutrition, poor oral health and falls. The process includes risk assessment, the analysis of causes of risks, the planning and performing of care interventions, and evaluations of the interventions [31]. A comprehensive preventive process, such as the one available in Senior Alert, is therefore crucial to support healthcare personnel in all forms of health care.

Because pressure ulcers, malnutrition, poor oral health and falls rarely develop in isolation, the focus should be on preventing health risks to enable healthy aging in older persons. However, most of the existing studies have investigated only one health risk at a time. In addition, these studies included specific patient populations, were conducted in a variety of settings and used a variety of assessment tools [32]. Thus, it is warranted to develop a comprehensive overview of the prevalence of risk for pressure ulcers, malnutrition, poor oral health and falls among older persons receiving municipal health care using the same instruments for each risk.

### Aim

The aim of this study was to determine the prevalence of risk for pressure ulcers, malnutrition, poor oral health and falls among older persons aged ≥65 years who receive municipal health care in southern Sweden.

## Methods

### Design

A retrospective cross-sectional study was conducted with data from the national quality register Senior Alert.

### Participants

The inclusion criteria for the study were older persons who were risk-assessed in Senior Alert for the first time for the risk of pressure ulcers, malnutrition, poor oral health and falls at age ≥ 65 years and who lived in a county in southern Sweden where they received municipal health care.

### Data collection

Based on the instruments available in Senior Alert (see below), risk assessments and registrations in Senior Alert were performed by healthcare personnel. Data were extracted from Senior Alert between 2018–08–01 and 2019–07–30. Data on the type of housing, age, body mass index (BMI), municipality and biological sex were also gathered from Senior Alert.

### Instruments available in Senior Alert

The Modified Norton Scale (MNS) was used to assess the risk of pressure ulcers [33], the short form of the Mini Nutritional Assessment (MNA-SF) was used to assess the risk of malnutrition or malnourishment [34], the Revised Oral Assessment Guide-Jönköping (ROAG-J) was uses to assess oral health [35, 36] and the Downtown Fall Risk Index (DFRI) [37] was used to assess the risk of falls.

### Definitions

#### Type of housing

##### Short term nursing homes

A shorter stay facility for older persons at special municipal residential care homes, which offer rehabilitation, aftercare, diagnosis or assessment of needs.

##### Home health care

Receiving health care in one’s own home.

##### Nursing homes

Receiving municipal health care in residential care homes.

##### Dementia care units

Receiving municipal health care in residential care homes for older persons with dementia diagnosis.

For further definitions, please see the Supplementary File.

### Statistical analysis

IBM SPSS Statistics for Windows, Version 25.0. (Armonk, NY: IBM Corp) was used for all the analyses. The prevalence of risk for pressure ulcers, malnutrition, poor oral health and falls was calculated using categorical data from the instruments that were extracted from Senior Alert (risk/no risk). To describe the study sample, descriptive analyses (i.e., percentage, frequencies, range, mean and standard deviation [SD]) were performed. T-tests to compare groups, chi-square tests were used to compare proportions, and the Mantel- Haenszel test to test trends was performed. Bonferroni correction for multiple comparisons was calculated when three or more comparisons were made. Bivariate and multivariate logistic regression models were performed to identify associations of the risk for pressure ulcers, malnutrition, poor oral health and falls and with demographic variables as independent variables. If independent variables were significantly associated with the health risks, they were then included in the multivariate logistic regression models. Association between these variables and health risks are expressed as odds ratio (OR) with 95% confidence intervals (CI). A *p*-value <.05 was considered to be statistically significant.

## Results

### Demographics

The study sample consisted of 12,518 persons ≥65 years who were registered as having risk or no risk based on the instruments used in Senior Alert, with a majority of women (*n* = 8265, 66.0%) (Table 1). Among older persons living in some type of care home, nursing homes were most common (*n* = 8558, 68.4%) (Table 1). In the total sample, the ages ranged from 65 to 106 years, with a mean age of 86 years (SD 7.7). The women were older than the men (mean 87.2 SD 7.3 vs. 83.6 SD 7.9 and *p* < 0.001). The mean age among the older persons in short term nursing homes was 84 years (SD 7.3), ranging from 65 to 102 years. For older persons receiving home health care, mean age was 84.5 years (SD 7.8), ranging from 65 to 106 years. In nursing home, the age ranged from 65 to 106 years, and the mean age was 86.6 years (SD 7.8). For older persons living in dementia care units, the mean age was 85.0 years (SD 7.3), and the ages ranged from 65 to 103 years. The mean BMI was 25.2 (SD 2.3) in the total sample, ranging from 11 to 63.

**Table 1:** Total sample overview (*n* = 12,518)

	Total sample ***n*** (%)	Women ***n*** (%)	Men ***n*** (%)
**Sex**	12,518 (100)	8265 (66.0)	4253 (34.0)
**Type of housing**			
**Short term nursing care**	487 (3.9)	269 (3.3)	218 (5.1)
**Home health care**	1692 (13.5)	1080 (13.1)	612 (14.4)
**Nursing homes**	8558 (68.4)	5763 (69.7)	2795 (65.7)
**Dementia care units**	1781 (14.2)	1153 (14.0)	628 (14.8)
**Total**	12,518 (100)	8265 (100)	4253 (100)
**Age groups (years)**			
**65–74**	1152 (9.2)	500 (6.0)	652 (15.3)
**75–84**	3666 (29.3)	2116 (25.6)	1550 (36.4)
**85–94**	6114 (48.8)	4372 (52.9)	1742 (41.0)
**95–106**	1586 (12.7)	1277 (15.5)	309 (7.3)
**Total**	12,518 (100)	8265 (100)	4253 (100)
**BMI**			
**< 18.5 (underweight)**	933 (7.5)	738 (8.9)	195 (4.6)
**18.5–24.9 (normal weight)**	5639 (45.0)	3705 (44.8)	1934 (45.5)
**25.0–29.9 (overweight)**	3853 (30.8)	2403 (29.1)	1450 (34.1)
**> 30.0 (obese)**	2080 (16.6)	1408 (17.0)	672 (15.8)
**Total**	12,505 (100)	8254 (100)	4251 (100)
**Municipalities**			
**Large cities/municipalities near large cities**	2959 (23.6)	1969 (23.8)	990 (23.3)
**Medium-sized towns/municipalities near medium-sized towns**	5817 (46.5)	3855 (46.6)	1962 (46.1)
**Smaller towns/urban areas/rural municipalities**	3742 (29.9)	2441 (29.5)	1301 (30.6)
**Total**	12,518 (100)	8265 (100)	4253 (100)

### Prevalence of risk for pressure ulcers, malnutrition, poor oral health and falls

In the total sample, the prevalence of risk for pressure ulcers, malnutrition, poor oral health and falls was 27.9, 56.3, 34.2 and 74.5% respectively. More detailed information about the prevalence of the health risks in relation to type of housing, age groups, BMI, municipalities and sex is presented in Table 2. For instance, Table 2 demonstrates, that the prevalence of risk for pressure ulcers, malnutrition and poor oral health was significantly higher among older persons with a BMI < 18.5 than among older persons with BMI > 18.5. The odds of having a risk for malnutrition were higher in the short term nursing care compared to other types of housing. The oldest age group of 95–106 years had the highest odds of having a risk for falls. The odds of having risk for poor oral health decreased with the size of the municipality. Female sex increased the odds of having a risk for pressure ulcers and risk for malnutrition, while it decreased the odds of having poor oral health (Table 3).

**Table 2:** Prevalence of risk for pressure ulcers, malnutrition, poor oral health and falls in according to type of housing, age groups, BMI, municipalities and sex (*n* = 12,518)

**Type of housing**	**Short term nursing care** *n* = 487	**Home health care** *n* = 1692	**Nursing homes** *n* = 8558	**Dementia care units** *n* = 1781	***P*** **values** < 0.05*
Risk of pressure ulcers	139 (28.5)	233 (13.8)	2573 (30.1)	553 (31.0)	A, D, E
Risk of malnutrition	413 (84.8)	801 (47.3)	4715 (55.1)	1115 (62.6)	A, B, C, D, E, F
Risk of poor oral health	152 (31.2)	470 (27.8)	2906 (34.0)	751 (42.2)	C, D, E, F
Risk of falls	375 (77.0)	1078 (63.7)	6448 (75.3)	1428 (80.2)	A, D, E, F
**Age groups**	**65–74** *n* = 1152	**75–84** *n* = 3666	**85–94** *n* = 6114	**95–106** *n* = 1586	P values**
Risk of pressure ulcers	265 (23.0)	1099 (30.0)	1656 (27.1)	478 (30.1)	0.070
Risk of malnutrition	601 (52.2)	2182 (60.0)	3374 (55.2)	887 (56.0)	0.506
Risk of poor oral health	472 (41.0)	1299 (35.4)	1992 (32.6)	516 (32.6)	< 0.001
Risk of falls	764 (66.3)	2699 (73.6)	4584 (75.0)	1285 (81.0)	< 0.001
BMI****	**< 18.5** *n* = 933	**18.5–24.9** *n* = 5639	**25.0–29.9** *n* = 3853	**> 30.0** *n* = 2080	P values**
Risk of pressure ulcers	465 (49.8)	1647 (29.2)	916 (23.8)	465 (22.4)	< 0.001
Risk of malnutrition	933 (100)	3739 (66.3)	1605 (41.7)	759 (36.5)	< 0.001
Risk of poor oral health	441 (47.3)	1956 (34.7)	1225 (31.8)	656 (31.5)	< 0.001
Risk of falls	706 (75.7)	4173 (74.0)	2880 (74.7)	1561 (75.0)	0.643
**Municipalities**	**Large cities/municipalities near large cities** *n* = 2959	**Medium-sized towns/municipalities near medium-sized towns** *n* = 5817	**Smaller towns/urban areas and rural municipalities** *n* = 3742		P values**
Risk of pressure ulcers	874 (29.5)	1628 (28.0)	996 (26.6)		0.008
Risk of malnutrition	1773 (59.9)	3254 (55.9)	2017 (53.9)		< 0.001
Risk of poor oral health	961 (32.5)	1973 (33.9)	1345 (35.9)		0.003
Risk of falls	2298 (77.7)	4306 (74.0)	2725 (72.8)		< 0.001
**Sex**	**Women** *n* = 8265	**Men** *n* = 4253			***P*** **values** ***
Risk of pressure ulcers	2413 (29.2)	1085 (25.5)			< 0.001
Risk of malnutrition	4704 (56.9)	2340 (55.0)			0.044
Risk of poor oral health	2733 (33.1)	1546 (36.4)			< 0.001
Risk of falls	6177 (74.7)	3152 (74.1)			0.449

Note: A=Short term nursing care compared to Home health care; B=Short term nursing care compared to Nursing homes; C=Short term nursing care compared to Dementia care units; D=Home health care compared to Nursing homes; E=Home health care compared to Dementia care units; F=Nursing homes compared to Dementia care units. *Chi square test with the Bonferroni correction for multiple analyses. All the presented p values were *p* < 0.006. Significant level in the current study was set at *p* < 0.05. **Test for trend. ***Chi square test. **** BMI was available for 99.9% of the measurements (*n* = 12,505)

**Table 3:** Demographic variables in relation to risk for pressure ulcers, malnutrition, poor oral health and falls analysed by multivariate logistic regression and expressed as odds ratio (OR) with 95% confidence intervals (CI)

Independent variables		Dependent variables			
Variables	Categories	Risk of pressure ulcers	Risk of malnutrition	Risk of poor oral health	Risk of falls
Type of housing	Home health care	Reference	Reference	Reference	Reference
	Short term nursing care	**2.50 (1.95–3.17)**	**6.10 (4.64–7.90)**	1.21 (0.97–1.51)	**1.84 (1.45–2.33)**
	Nursing homes	**2.68 (2.31–3.10)**	**1.32 (1.20–1.48)**	**1.41 (1.25–1.58)**	**1.65 (1.47–1.84)**
	Dementia care units	**2.79 (2.35–3.32)**	**1.83 (1.59–2.09)**	**1.96 (1.70–2.26)**	**2.24 (1.92–2.61)**
Age groups (years)	65–74	Reference	Reference	Reference	Reference
	75–84	**1.37 (1.17–1.60)**	**1.31 (1.15–1.51)**	**0.79 (0.69–0.91)**	**1.44 (1.20–1.61)**
	85–94	1.13 (0.98–1.32)	1.10 (0.98–1.25)	**0.70 (0.62–0.80)**	**1.49 (1.30–1.71)**
	95–106	**1.25 (1.05–1.50)**	1.15 (0.99–1.35)	**0.71 (0.61–0.84)**	**2.09 (1.75–2.49)**
Municipalities	Smaller towns/urban areas and rural municipalities	Reference	Reference	Reference	Reference
	Medium-sized towns/municipalities near medium-sized towns	0.99 (0.90–1.09)	**1.11 (1.02–1.20)**	**0.91 (0.83–0.99)**	1.04 (0.94–1.14)
	Large cities/municipalities near large cities	1.05 (0.94–1.17)	**1.19 (1.08–1.32)**	**0.83 (0.75–0.91)**	**1.21 (1.08–1.36)**
Sex	Men	Reference	Reference	Reference	–
	Women	**1.20 (1.10–1.30)**	**1.11 (1.03–1.20)**	**0.90 (0.83–0.98)**	–

Note: Significant risk factors are depicted in bold

### Number of risks

**Fig. 1:**
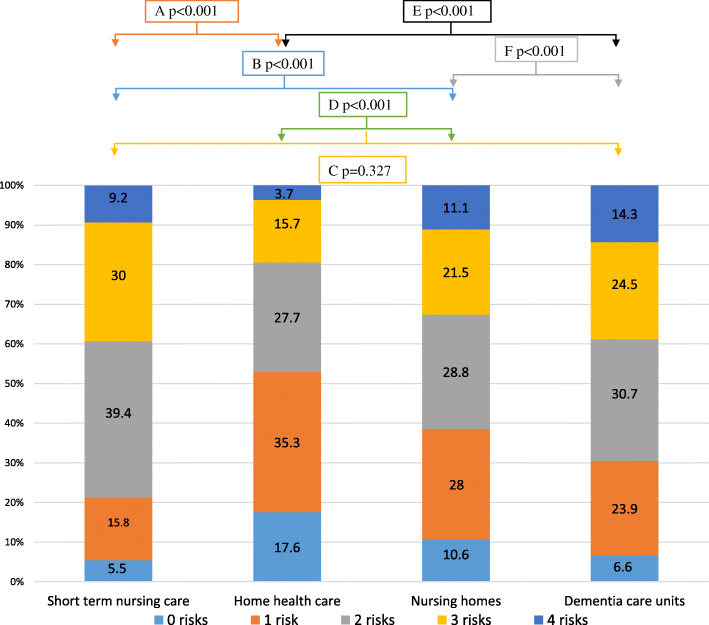
Prevalence reported in percentages of multiple risks distributed by type of housing (*n* = 12,518). Note: A=Short term nursing care compared to Home health care; B=Short term nursing care compared to Nursing homes; C=Short term nursing care compared to Dementia care units; D=Home health care compared to Nursing homes; E=Home health care compared to Dementia care units; F=Nursing homes compared to Dementia care units. Test for trend

**Fig. 2:**
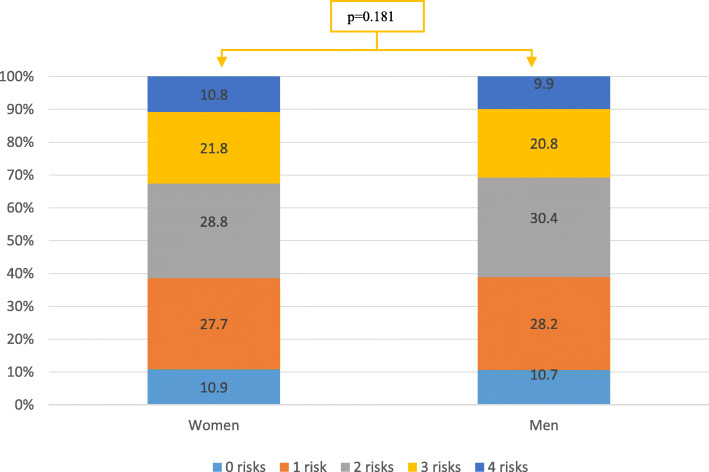
Prevalence (in percentages) of multiple risks among women and men (*n* = 12,518). Test for trend

In the total sample, approximately 90% of the older persons had at least one of the health risks, almost 30% had two health risks, over 21% had three health risks and approximately 11% had all four health risks. As presented in Fig. 1, the presence of multiple health risks in one subject was more common in dementia care units compared to nursing homes and home health care but not compared to short-term nursing care. There was no significant difference in the presence of multiple risks between women and men (Fig. 2).

## Discussion

Alarmingly, our results show that 90% of individuals had at least one health risk and every tenth older person in this study had four health risks. Having four health risks was more common among older persons in dementia care units. Because an increase in age is the most important risk factor for dementia and as demographic aging occurs worldwide, more people are expected to develop dementia [38]. Therefore, our results highlight the high demands that will likely be placed on healthcare personnel working within dementia care units. Almost 30% of the older persons in the current study had two health risks, indicating that focusing on preventing only one health risk may not be sufficient [39]. As pressure ulcers, malnutrition, poor oral health and falls are multifactorial and related to frailty [4], the focus should be on preventing all of these health risks. Since most older persons living in nursing homes are frail [40] and several risk factors are mutual for all the health risks, working according to the preventive process used in Senior Alert might improve older persons’ health status [31]. This process includes adequate prevention care interventions that target multiple risk factors and has been proven to be effective [41].

The prevalence of risk for pressure ulcers among older persons in short term nursing homes (29%) and dementia care units (31%) in our study is in line with a study conducted in a Swedish context (26 and 35% respectively) [42], but not with a systematic review conducted in a German context (2–5%) [43]. Old age and female sex were associated with the risk of pressure ulcers, which is in line with a study conducted in the Netherlands [6]. This suggests that women aged 65 years and over are especially vulnerable to the risk for pressure ulcers. The prevalence of risk for malnutrition in nursing homes was 55% compared to 40–62% in studies conducted in other Swedish nursing homes [18, 22, 44] and 15–40% in nursing homes internationally [19–21]. In our study, the prevalence of risk for malnutrition increased among older persons living in short terms nursing homes. Since cognitive impairment, low mood, medications and poor oral health may occur due to acute illness requiring hospital admission [45] and because nutritional status is estimated to worsen during a hospital admission [46], older persons transferred to short term nursing homes (to recover) may already be frail [47]. Overall, this emphasizes the importance of the preventive work regarding malnutrition and pressure ulcers, which is highlighted by The European Society for Clinical Nutrition and Metabolism (ESPEN) [48].

Given that poor oral health is associated with dementia [49], oral treatment among older persons with cognitive impairments may be perceived as a violation of integrity and could explain why over 40% of older persons in dementia care units had a risk of poor oral health in the current study. However, our results demonstrate that the risk of poor oral health decreased with increasing age, which could be because older persons in the youngest age group are expected to manage their oral health without assistance from healthcare personnel.

The high risk of falls (77%) among older persons staying in short term nursing homes is similar to a Swedish study (79%) [50]. However, the prevalence of risk for falls (80%) among older persons in dementia care units was higher compared to a study conducted in United Kingdom (66%) [51]. The prevalence of risk for falls increased with age, which is in line with previous research highlighting age as a significant risk factor for falling [52]. Frailty increases with age [53], which could explain our result.

BMI standard classification is not well suited to assess older persons [54] and the Global Leadership Initiative on Malnutrition (GLIM) discuss other cutoffs for older persons regarding BMI (< 20 if < 70 years or < 22 if > 70 years) [55]. Importantly, efforts to ensure that older persons do not lose weight should be prioritized, because weight loss is strongly associated with frailty and has a negative impact on health outcomes [40].

The stay-at-home policy in Sweden offers home health care for as long as is needed, and as a consequence, older persons moving in to care homes are frailer [56]. This is in line with our study suggesting that preventive work is particularly crucial in care homes. Additionally, the lack of specialist nurses in the field of elderly care in Sweden suggests the need for increased knowledge in this area. To address the challenges associated with demographic changes, one clinical implication could be to educate healthcare personnel in elderly care. Therefore, we suggest a pedagogical intervention aimed at increasing the knowledge of nurses, nurse assistants and managers in municipal health care. This intervention should focus on the entire preventive process to reduce the risk of pressure ulcers, malnutrition, poor oral health and falls.

One strength of this current study is the large sample of older persons receiving different kinds of municipal health care. The large sample could be considered as representative, thus strengthening the generalizability of our findings. However, one must bear in mind that the data were extracted over a one-year period, and therefore, prevalence studies must be interpreted carefully, as they reflect only a snapshot of reality. Another strength is that the instruments used for Senior Alert are well known. Despite this and that the instruments are considered useful in routine practice without any expert competence, the users may interpret and use the instruments differently, resulting in differing results. The possibility of incorrect risk assessments could be a limitation of this study.

## Conclusion

The prevalence of the risk for pressure ulcers, malnutrition, poor oral health and falls is still high in southern Sweden. As many as 90% of older persons in this study had at least one health risk. Older persons living in dementia care units in particular had a high risk of having several health risks. To reduce the risk of pressure ulcers, malnutrition, poor oral health and falls in older persons receiving municipal health care, a continued focus must be placed on increasing knowledge of a comprehensive preventive process for healthcare personnel to meet the challenges facing the aging demographic.

## Supplementary Information

**Additional file 1.**

## Data Availability

The data that support the findings of this study are available from Senior Alert but restrictions apply to the availability of these data, which were used under license for the current study, and are not publicly available.
