# Supplementary material for: Prevalence of risk for pressure ulcers, malnutrition, poor oral health and falls – a register study among older persons receiving municipal health care in southern Sweden
Source: BMC Geriatr. 2021 Apr 21;21:265. doi: 10.1186/s12877-021-02205-x (PMC8059027; doi:10.1186/s12877-021-02205-x)
Supplement: Supplementary file 1 — Additional file 1. [file 12877_2021_2205_MOESM1_ESM.docx]

**Supplementary File**

Definitions

Body mass index, BMI, (the weight in kilograms divided by the square of the height in meters [kg/m2]) is categorized as:

*<18.5* underweight

*18.5*–*24.9* normal weight

*25.0*–*29.9* overweight

*>30* obese

The municipalities were defined in accordance with the Swedish Association of Local Authorities and Regions (SALAR) (Classification of Swedish municipalities 2017).

*Large cities:* at least 200 000 inhabitants in the largest urban area and *municipalities near large cities:* at least 40 % of the working population commute to work in a large city or municipality near a large city.

*Medium-sized towns:* minimum 40 000 and maximum 199 999 inhabitants in the largest urban area and *municipalities near medium-sized towns;* municipalities where more than 40 % of the working population commute to work in a medium-sized town and municipalities where less than 40% of the working population commute to work in a medium-sized town.

*Smaller towns/urban areas:* minimum 15 000 and maximum 39 999 inhabitants in the largest

urban area and *rural municipalities*: fewer than 15 000 inhabitants in the largest urban area,

very low commuting rate (less than 30%).
